# A Hybrid SVR-Based Prediction Model for the Interfacial Bond Strength of Externally Bonded FRP Laminates on Grooves with Concrete Prisms

**DOI:** 10.3390/polym14153097

**Published:** 2022-07-29

**Authors:** Kaffayatullah Khan, Mudassir Iqbal, Rahul Biswas, Muhammad Nasir Amin, Sajid Ali, Jitendra Gudainiyan, Anas Abdulalim Alabdullah, Abdullah Mohammad Abu Arab

**Affiliations:** 1 Department of Civil and Environmental Engineering, College of Engineering, King Faisal University, Al-Ahsa 31982, Saudi Arabia; mgadir@kfu.edu.sa (M.N.A.); 218038024@student.kfu.edu.sa (A.A.A.); 219041496@student.kfu.edu.sa (A.M.A.A.); 2 Department of Civil Engineering, University of Engineering and Technology, Peshawar 25120, Pakistan; mudassiriqbal@uetpeshawaitr.edu.pk; 3 Department of Applied Mechanics, Visvesvaraya National Institute of Technology Nagpur, Nagpur 440010, India; rahulbiswas@apm.vnit.ac.in; 4 Mechanical and Energy Engineering, College of Engineering, Imam Abdulrahman Bin Faisal University, P.O. Box 1982, Dammam 31451, Saudi Arabia; sakzada@iau.edu.sa; 5 Department of Civil Engineering, GLA University, Mathura 281406, India; jitendra.gudainiyan@gla.ac.in

**Keywords:** interfacial bond strength, fiber-reinforced polymer, single-lap shear test, support vector machine, meta-heuristic optimization algorithms

## Abstract

The current work presents a comparative study of hybrid models that use support vector machines (SVMs) and meta-heuristic optimization algorithms (MOAs) to predict the ultimate interfacial bond strength (IBS) capacity of fiber-reinforced polymer (FRP). More precisely, a dataset containing 136 experimental tests was first collected from the available literature for the development of hybrid SVM models. Five MOAs, namely the particle swarm optimization, the grey wolf optimizer, the equilibrium optimizer, the Harris hawks optimization and the slime mold algorithm, were used; five hybrid SVMs were constructed. The performance of the developed SVMs was then evaluated. The accuracy of the constructed hybrid models was found to be on the higher side, with R^2^ ranges between 0.8870 and 0.9774 in the training phase and between 0.8270 and 0.9294 in the testing phase. Based on the experimental results, the developed SVM–HHO (a hybrid model that uses an SVM and the Harris hawks optimization) was overall the most accurate model, with R^2^ values of 0.9241 and 0.9241 in the training and testing phases, respectively. Experimental results also demonstrate that the developed hybrid SVM can be used as an alternate tool for estimating the ultimate IBS capacity of FRP concrete in civil engineering projects.

## 1. Introduction

As a result of the corrosion of traditional steel reinforcement, reinforced concrete (RC) structures are required to undergo regular maintenance and repair [1]. Therefore, strengthening existing structures is regarded as an increasing priority in the construction industry, as it is necessary in order to comply with improved code designs and strength criteria [2]. Hence, fiber-reinforced polymer (FRP) laminates are increasingly being employed for the purpose of retrofitting and improving the current structural capacity of beams [3,4,5], columns [6,7] and beam–column junctions [8,9,10,11] due to their high performance.

FRP plates may potentially replace steel plates in structural reinforcement due to their light weight and high strength, as well as their superior resistance to corrosion [12], creep/fatigue [13] and hygrothermal stresses [14]. However, notwithstanding those benefits, FRP incorporation has some disadvantages, such as rupture, concrete crushing, shear cracks and debonding between concrete and laminates that propagates through FRP-strengthened structures and causes more damage [15,16,17]. FRP rupture or concrete crushing may occur if the ends of the reinforcing plates are not properly anchored [18]. Even before reaching full capacity, premature debonding can be observed and is the most common recorded failure; it leads to the debonding of the FRP laminate and progresses towards the center from the ends [19]. The diffusion of water molecules into the FRP plate may produce irreversible interfacial debonding, resulting in a reduction in the interlaminar shear strength of the FRP plate. Increased temperatures can also aggravate debonding [14]. There may be interfacial FRP debonding as a result of dynamic loads and thermal aging [20]. Strengthened constructions lose structural capacity when FRP laminates debond from one another, and this is a serious problem [16]. In FRP-strengthened RC members, early plate debonding from the concrete prism has been found in experiments. The composite action between an FRP component and a concrete prism determines the failure model of a reinforced component. Plate-end debonding and intermediate crack-induced debonding may occur if the composite process between the FRP and the concrete continues [21]. Interfacial bond failures can also be caused by poor bond quality due to poor workmanship [22].

**Figure 1:**
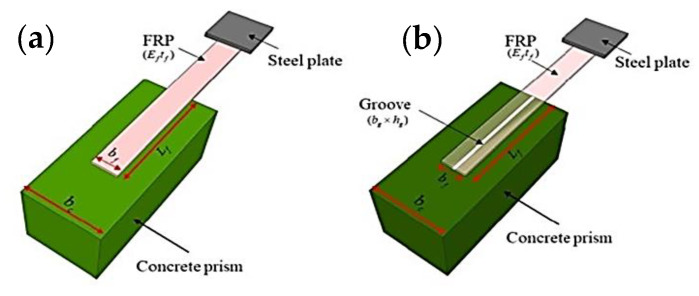
Single-lap shear test: (**a**) FRP externally bonded on concrete; (**b**) FRP externally bonded on the grooves of concrete (adapted with permission from Su et al. [58]).

Existing laboratory studies have demonstrated that improper preparation of the concrete-to-FRP interface is the most significant cause of early failure in the form of FRP debonding [23]. It is possible to increase the strength of the bond between FRP and concrete in several ways, including epoxy interlocking near the surface mounting. The need for a flexible interface between concrete and FRP laminate for the flexural strengthening of a beam has therefore been identified as a key requirement. Removing the damaged surface layer of the concrete and exposing the coarse aggregate is a part of the surface preparation process. As a result, the final rupture strength is increased due to the delayed debonding of the FRP sheet from the concrete surface. The concrete is sandblasted, the dust is removed with a brush and solvents are used to clean the surface; then, the FRP sheets are installed. Surface mounting techniques are used for FRP strengthening; these include FRP rebars and laminates placed in grooves and packed with high-adhesive materials [24,25,26,27]. Another option is to use FRP laminates that are externally bonded to the concrete’s surface in grooves (Figure 1b). The surface area, material availability, cost, safety, and need for supporting equipment are the major factors that affect the bond [28]. To quantify interfacial bond strength (IBS), some basic experimental approaches, such as the single-lap shear test (SST), have been utilized because of their reliability and simplicity [29,30,31]. Previous research has led to the development of empirical and semi-empirical formulations for predicting IBS based on experimental data from SSTs [17,32,33]. These formulations have been found to be reasonably accurate. Despite the fact that the available models’ empirical relations have a good level of congruence with the experimental data, these models have not been validated using more recent data. In addition, in order to construct these empirical relations, some fundamental simplifications and assumptions were made [34].

Hence, an alternative method of modeling was needed that could replace those complex empirical relations and give more accurate results. Hence, the application of artificial intelligence came into use. The term “artificial intelligence” (AI) refers to the widespread application of computer programming techniques to solve complex engineering issues, particularly those involving regression and classification [35,36,37,38,39,40,41,42,43,44,45,46,47,48,49,50,51,52,53]. AI models are not only trained by using a large number of experimental observations but also validated using a new dataset [44,54]. In addition, there are several successful applications of AI models in the field of composite construction. According to Vu and Hoang [55], the least square support vector machine was able to forecast the punching shear capacity of FRP-reinforced concrete beams with a coefficient of determination (R^2^) equal to 0.99. An artificial neural network (ANN) was utilized by Hoang [56] to forecast the punching shear capacity of steel-fiber-reinforced concrete slabs. Research by Abuodeh et al. [57] used neural interpretation diagrams (NIDs) and recursive feature elimination (RFEs) to analyze the shear capability of RC beams.

Finally, in order to improve the efficiency of engineering projects, AI models based on available experimental data are needed to estimate the IBS of FRP plates attached to a concrete prism. Su et al. [58] developed multilinear regression and ANN AI models to forecast the IBS of FRP laminates to a concrete prism for two different cases; without groove (Figure 1a) and with groove (Figure 1b), namely. The training and validation data yielded R^2^ values of 0.81 and 0.91, respectively. However, an ANN’s inability to define any meaningful relation between model input and output is a fundamental obstacle to its successful deployment in real-world applications [59,60]. Over-fitting and local minimum problems are two further issues that arise while using ANNs [61]. ANNs also have certain inherent limitations. On the other hand, Vapnik invented support vector machines (SVMs), a new artificial intelligence technique that uses statistical learning theory to solve structural engineering issues [62]. SVMs employ the structured risk minimization (SRM) concept, which outperforms the standard empirical risk minimization (ERM) principle used by conventional neural networks in terms of generalization performance [63,64,65,66,67]. The number of local minima, the sparseness of the solution and the number of support vectors are all important parameters in SVMs. Hence, it has been concluded from past studies that the use of hybrid SVMs is a new approach in this area; in the present work, using support vector regression, an attempt was made to forecast the IBS of FRP laminates. Through the use of 136 different experimental SST results, this study investigated the extent to which the SVR model is able to estimate the IBS of FRP laminates that are externally connected to a concrete prism using grooves (the anchorage made on one end of an FRP component to a concrete prism is shown in Figure 1b). In the analysis, we used samples that had been tested using FRP plates aligned in a direction parallel to the groove. On top of that, the parametric analysis and visual interpretation (Taylor diagram) are shown to demonstrate how the input factors affect IBS.

## 2. Methodology

### 2.1. Overview of Optimization Algorithms

**Figure 2:**
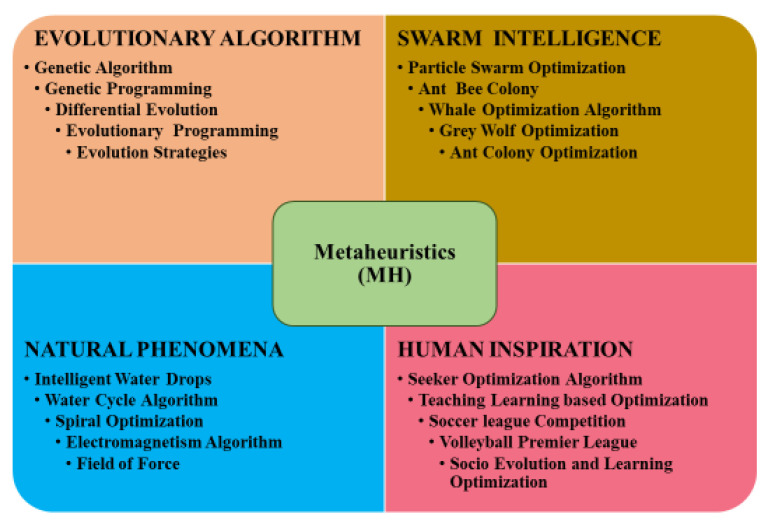
Metaheuristic model classification.

In this part, meta-heuristic approaches are investigated. In engineering, the use of meta-heuristic optimization algorithms (MOAs) to tackle a variety of issues has grown substantially. They are free gradient methods that can solve highly challenging optimization problems more effectively than conventional approaches [68]. In addition, they are simpler and faster to implement than conventional optimization methods [69]. There are numerous inspiration sources for MOAs, which can be categorized into distinct groupings based on these inspiration sources. Among these groups are evolutionary algorithms (EAs), swarm intelligence (SI) methodologies, natural phenomenon approaches and human-inspired algorithms [70,71]. Figure 2 illustrates these divisions. The purpose of the first group of algorithms, known as EAs, is the simulation of natural genetic processes such as crossover, mutation, and selection. This category contains several MOAs, including evolutionary programming [72], evolutionary strategy (ES) [73], the equilibrium optimizer (EO), genetic algorithms [74], decision trees [75] and genetic programming (GP) [76]. The second group, SI, mimics the swarming behavior observed in nature when searching for food. The most notable algorithms in this area include the particle swarm optimization (PSO) [77], the artificial bee colony (ABC) [78], the grey wolf optimization (GWO) [79], the ant colony optimization (ACO) [80], the salp swarm algorithm (SSA) [81], the marine predators algorithm (MPA) [82], the Harris hawks optimization (HHO) [83], the slime mold algorithm [84] and the whale optimization algorithm (WOA) [85]. This category also includes the spiral optimization (SO) [86], the water cycle algorithm (WCA) [87], the intelligent water drop (IWD) [88], the field of force (FOF) [89] and the electromagnetism algorithm (EA) [90]. Furthermore, extra operations that adhere to physical rules fall under this group. As an illustration, this group includes the field of force (FOF) [91], the electromagnetism algorithm [92], the charged system search (CSS) [93], the gravitational search algorithm (GSA) [94], simulated annealing [95], the aquila optimizer (AO) [96], the electromagnetism-like mechanism, the flow regime algorithm (FRA) [97], the charged system search (CSS) [98], the optics-inspired optimization (OIO) [99] and the chemical reaction. In addition, human activities affect the fourth category [100]. This category includes algorithms such as the teaching–learning-based optimization (TLBO) [101], the volleyball premier league algorithm (VPL) [102], the soccer league competition (SLC) [103], the seeker optimization algorithm (SOA) [104], the league championship algorithm (LCA) [105] and the socio-evolution and learning optimization (SELO) [106].

Five distinct SI algorithms, namely PSO, GWO, EO, HHO and SMA, were employed to generate hybrid SVM models in this study. A brief discussion of these OAs is provided in the subsections that follow. This section provides a theoretical background and a short discussion of PSO, GWO, EO, HHO and SMA. Subsequently, the methodological development of hybrid SVMs is presented and discussed. However, before presenting the above details, the working principle of SVMs is briefly presented.

### 2.2. Support Vector Machines (SVMs)

A Support vector machine (SVM) is a supervised machine learning method that may be used for both regression and classification. It was created by Vapnik in 1995 [107] and is based on statistical learning theory. The SVM technique projects data into a high-dimensional feature space and employs kernels to classify nonlinearly separable datasets [108,109]. In multidimensional space, an SVM model is essentially a representation of various classes in a hyperplane. The SVM generates the hyperplane in an iterative manner in order to reduce errors. The SVM’s objective is to split datasets into classes such that a maximum marginal hyperplane (MMH) may be found. The data points closest to the hyperplane, or the points of a dataset that, if deleted, would change the location of the dividing hyperplane, are called support vectors. As a result, they may be regarded as important components of a collection of data. In general, the accuracy of the SVR model is determined by the kernels used and their parameters. The radial basis function (RBF) has been shown to perform well as a kernel function for SVMs in several forecasting experiments [110,111,112].

For a dataset $\omega =\left\{\left(x_{i},y_{i}\right)i=1,2\ldots n\right\}$ where $x\in R^{d}$ is a d-dimensional input vector space, and y∈R is an output in a one-dimensional vector space, SVM regression can estimate the relationship between x and y. In the SVM approach, the risk function is minimized by minimizing both empirical risk and $\|\omega {\|}^{2}$.

(1) $$R=\frac{1}{2}\|\omega {\|}^{2}+C_{C}\underset{i=1}{\sum }l_{\varepsilon }(y_{i}-f\left(\underset{x_{i}}{\to }\right))$$

where the regression data vector is ‖ω‖, and loss is denoted by $l_{\varepsilon }$, which presents the difference between $y_{i}$ (real output) and $f\left(\underset{x_{i}}{\to }\right)$. A positive constant value $C_{C}$ is needed to fix the prior. $l_{\varepsilon }\left(y_{i}-f\left(\underset{x_{i}}{\to }\right)\right)\;$ is 0 for $y_{i}-f\left(\underset{x_{i}}{\to }\right)<\epsilon$. Otherwise, it is equal to $y_{i}-f\left(\underset{x_{i}}{\to }\right)\;$. Minimizing the risk function can be accomplished with the following function:

(2) $$f\left(x,\alpha ,\alpha^{*}\right)=\overset{l}{\underset{i=1}{\sum }}\left(\alpha_{i}^{*}-\alpha_{i}\right)\left(\varphi \left(x_{i}\right),\varphi \left(x\right)\right)+b$$

where $\alpha_{i}^{*}.\alpha_{i}=0$ and $\alpha_{i}^{*}.\alpha_{i}\geq 0$; $\varphi \left(x_{i}\right),\varphi \left(x\right)$ is a product of the kernel function, and b is a bias term [111].

### 2.3. Particle Swarm Optimization (PSO)

It was Kennedy and Eberhart [113] who first introduced PSO to the scientific community as part of the swarm-based community in 1995. It is the primary goal of PSO to find global optimal solutions in a multidimensional setting. PSO begins by implementing the random speeds and locations of objects. Next, each object adjusts its position to pick the appropriate status in a multidimensional environment based on its speed, personal best position and global best position. This process continues until the optimal solution is found. It has been determined that the best position that can be gained by individual particles is the ideal status on a global scale; nevertheless, the most desired alternative that can be obtained by the particle is the ideal position on a personal scale. The location of the particle shifts as a result of considering both its optimal personal position and the optimal orientation for its optimal global location. At the same time, the speeds of the objects are altered in accordance with the disparity that exists between their best personal and best global positions. The particles move closer and closer to the optimal location as a result of a combination of exploring and exploiting. The acceleration coefficients c_1_ (cognitive coefficient) and c_2_ (social coefficient), which have fixed values of 1 and 2, respectively, are dependent on the situation at hand and reflect the level of confidence an element possesses in comparison to its personal and global status. Previous studies [114] provide information regarding the PSO operating principle in greater depth.

### 2.4. Grey Wolf Optimization (GWO)

Grey wolf optimization is based on the rigid hierarchy of grey wolves’ hunting behavior [79]. An alpha (α) group, consisting of a small number of males and females, makes major decisions such as hunting and is considered the ideal solution. The second level of the pack, which makes choices and follows orders from the alpha wolves, is known as beta (β). When alphas die or are too old and must be replaced, the best candidate is a female beta. Delta (δ) wolves are the third level of wolves, and they serve as sentinels and scouts and are used in the hunt. Omega (ω), the final level of the pack, is considered the most vulnerable and is tasked with keeping an eye on the young wolves. Grey wolf hunting was described by Muro et al. [115] in three stages: (a) recognizing, following and closing in on the target; (b) encircling the target; and (c) charging the target. These distinct social behaviors are treated by the GWO algorithm as separate variables to consider. A good starting point for this algorithm’s modeling stage is alpha, followed by beta, delta and omega. Detailed information about GWO can be found in Mirjalili et al. [79].

### 2.5. Equilibrium Optimizer (EO)

Faramarzi et al. [75] were the first to present an EO algorithm based on dynamic mass balance. The concentration of a nonreactive component in a control volume can be determined using various source and sink methods according to the EO methodology. For the preservation of mass entering, leaving and producing, mass balance equations are essential. Every particle (solution) in EO is a search agent, and its concentration (position) determines how effective the search is. To achieve equilibrium, the search agents randomly adjust their concentration to the best-so-far solutions, namely the equilibrium candidates (optimal result). The ability of EO to conduct exploration, exploitation and local minima avoidance is supported by a well-defined concept of “generation rate”. The main advantage of EO is that it has a straightforward framework that is easy to implement.

### 2.6. Harris Hawks Optimization (HHO)

Using SI-based optimization, Heidari et al. [83] developed HHO, a method that relates the hunting habits of Harris hawks to computer systems. Attacking prey (typically rabbits) from multiple directions and employing dynamic and sophisticated strategies that adapt to the prey’s fleeing pattern results in exhausted, bewildered prey. There are three steps to the algorithm. An exploratory phase is the first step, in which the birds represent possible solutions; they chase the chosen challenge and make observations. The prey’s type and energy determine the second step, which is the transition from exploration to exploitation. In the third step, the identified prey is assaulted and besieged from all sides during the exploitation process. The energy level of the prey, which is determined in the second stage, determines the difficulty of the siege.

### 2.7. Slime Mold Algorithm (SMA)

Meta-heuristics are influenced by nature, such as with SMA [84], which was developed recently and incorporates mathematical simulations of slime mold propagation waves that determine the optimal path for connecting foodstuffs. Slime mold, a eukaryotic organism found in nature, uses multiple food sources simultaneously to build a venous network connecting said food sources; this mold has unique characteristics and patterns. Slime mold can reach a size of over 900 cm^2^ if it is provided with enough food. The bio-oscillator creates a spreading wave that boosts the cytoplasmic flow into the veins, resulting in thicker veins by increasing the pace of cytoplasmic flow. In light of both its positive and negative reactions, slime may serve as an optimal conduit for food interaction. Since the wave propagation of slime mold has been replicated mathematically through the use of path networks and graph theory, the code has also been modeled in this way. Slime molds can also alter their dynamic search patterns based on the quality of the food they eat. One level of the slime mold algorithm is based on the behavior of slime when acquiring food based on the smell of the air, and the other level is based on the behavior of slime when it executes the contraction of its venous structure when food is warped around it. The initial work by Li et al. [84] provides comprehensive information about SMA, including the basic theory behind it.

### 2.8. Hybridization Procedure for SVMs and OAs

SVR parameters must be properly defined in order for the model to be successfully implemented and for good performance to be achieved. It is necessary to discover the global optimal solution to attain the greatest possible performance in order to ensure the accuracy of the SVR model’s performance. This can be considered an optimization problem. The SVR model’s two key parameters (the regularization parameter (γ) and the penalty factor (C)) were found using metaheuristic techniques. Choosing the optimum SVM settings is not possible without additional data. Model identification (the search for parameters) is, therefore, an important step. The algorithms proposed here were evaluated based on the RMSE value in the training stage to predict unknown data with sufficient accuracy and with minimal error between the predicted and target variables. In the exponential space, the parameters γ and C were explored. Five hybrid models (SVM–PSO, SVM–GWO, SVM–EO, SVM–HHO and SVM–SMA) were created by combining the SVR model with the metaheuristic algorithms PSO, GWO, EO, HHO and SMA.

## 3. Data Processing and Analysis

### 3.1. Descriptive Statistics and Statistical Analysis

**Figure 3:**
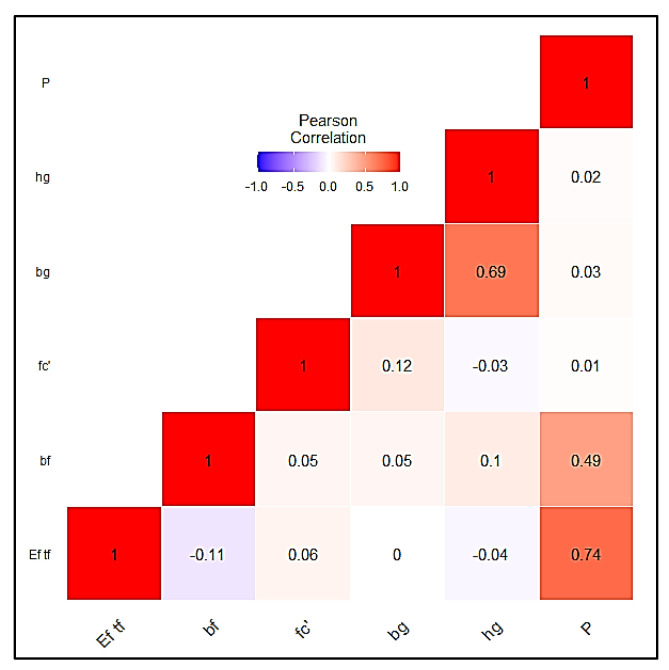
Pearson correlation with heat map.

A collection of 136 experimental results for the single-lap shear test was obtained from previous studies by Moghaddas and Mostofinejad [116], as reported by [58]; these results were used to develop a hybridized SVM model. The specimens were prepared such that FRP laminates were bonded to a concrete prism with the help of grooves, as shown in Figure 1b. Subsequently, the samples were subjected to the single-lap shear test. The elastic modulus of FRP multiplied by the thickness of the fiber (*E_f_ t_f_*, GPa-mm), which is also known as the axial stiffness; the width of the FRP (*b_f_*, mm); the concrete’s compressive strength (*fc*, MPa); the width of the groove (*b_g_*, mm) and the depth of the groove (*h_g_*, mm) were all been utilized as input variables, while the ultimate capacity (P, KN) was regarded a target variable to train the hybrid models. Table 1 shows the descriptive statistics of the input and output parameters, where it can be seen that the *E_f_ t_f_* varies from 12.90 to 78.20 with a skewness of 0.58, bf varies from 60 to 6270, *b_g_* and *h_g_* vary from 10 to 1405, *f_c_* varies from 48.20 to 4585.40 and the output value *p* varies from 4.76 to 25.49 with a skewness of 0.80; these values indicate the wide variety of experimental data. Statistical analysis was undertaken in order to measure the degree of correlation (DOC) using the Pearson correlation (Figure 3) between the above parameters after the descriptive analysis described above. Statistical analysis revealed that the collected database had a wide range of experimental data. When all parameters were evaluated, the DOCs between *p* and other parameters (excluding *E_f_ t_f_* and *b_f_*) were smaller, according to the information provided by the Pearson correlation in Figure 3. The DOCs between *p* and both *E_f_ t_f_* and *b_f_* were, on the other hand, shown to be significantly higher. Hence, the availability of a wide range of data, as seen from descriptive analysis, confirms that it can be utilized as an input parameter for the desired output.

### 3.2. Performance Parameters

Eight different performance indices (Equations (3)–(10)), namely the determination coefficient (R^2^), the performance index (PI), the variance account factor (VAF), Willmott’s index of agreement (WI), the root mean square error (RMSE), the mean absolute error (MAE), the RMSE observation standard deviation ratio (RSR) and the weighted mean absolute percentage error (WMAPE), were determined to evaluate the performance of the developed models [38,44,117,118,119,120,121,122,123,124,125,126,127,128,129,130,131,132,133]. For a flawless prediction model, the values of these indices should be identical to their ideal values, as shown in Table 2. Note that the generalization capacity of any predictive model is evaluated by determining various metrics, such as the degree of correlation, the associated error, the amount of variation, etc., from these diverse aspects.

(3) $$R^{2}=\frac{\sum_{i=1}^{n}{\left(y_{i}-y_{mean}\right)}^{2}-\sum_{i=1}^{n}{\left(y_{i}-{\hat{y}}_{i}\right)}^{2}}{\sum_{i=1}^{n}{\left(y_{i}-y_{mean}\right)}^{2}}$$

(4) $$\mathrm{PI}=adj.R^{2}+0.01\mathrm{VAF}-\mathrm{RMSE}$$

(5) $$\mathrm{VAF}\;\left(\%\right)=\left(1-\frac{var(y_{i}-{\hat{y}}_{i})}{var(y_{i})}\right)\times 100$$

(6) $$\mathrm{WI}=1-\left[\frac{\sum_{i=1}^{n}{\left(y_{i}-{\hat{y}}_{i}\right)}^{2}}{\sum_{i=1}^{n}{\left\{\left|{\hat{y}}_{i}-y_{mean}\right|+\left|y_{i}-y_{mean}\right|\;\right\}}^{2}}\right]$$

(7) $$\mathrm{RMSE}=\sqrt{\frac{1}{n}\sum_{i=1}^{n}{\left(y_{i}-{\hat{y}}_{i}\right)}^{2}}$$

(8) $$\mathrm{MAE}=\frac{1}{n}\sum_{i=1}^{n}\left|\left({\hat{y}}_{i}-y_{i}\right)\right|$$

(9) $$\mathrm{RSR}=\frac{\mathrm{RMSE}}{\sqrt{\frac{1}{n}\sum_{i=1}^{n}{\left(y_{i}-y_{mean}\right)}^{2}}}$$

(10) $$\mathrm{WMAPE}=\frac{\sum_{i=1}^{n}\left|\frac{y_{i}-{\hat{y}}_{i}}{y_{i}}\right|\times y_{i}}{\sum_{i=1}^{n}y_{i}}$$

where $y_{i}$ is the actual value, ${\hat{y}}_{i}$ is the predicted value and $y_{mean}$ is the mean of the actual value.

## 4. Results and Discussion

### 4.1. Parametric Configuration

**Figure 4:**
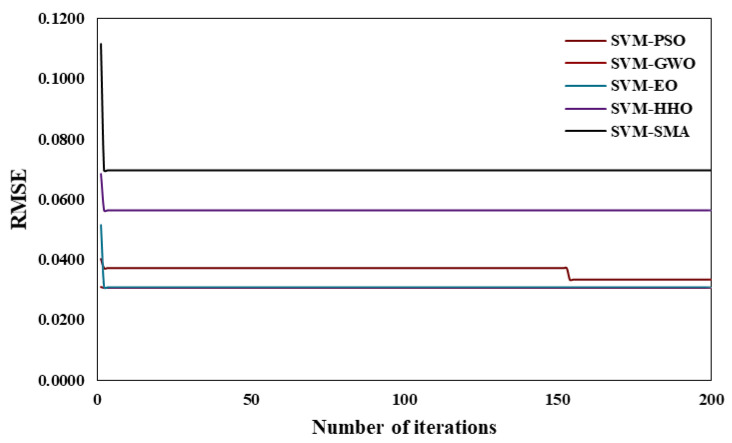
Convergence behavior of hybrid SVM models.

As mentioned earlier, to construct optimum hybrid models, it is necessary to prespecify the hyper-parameters of the SVM. The values of γ and C were set (using trial and error) as shown in Table 3 for different hybrid SVM models. Following a trial-and-error approach, the most appropriate values for the swarm size (N_S_) and the number of iterations (Itr) were set at 30 and 200, respectively, and were kept constant for other hybrid SVM models. It is also important to note that the convergence behavior of any OA is essential when evaluating performance. This is because the convergence behavior exposes the ability of OAs to break out of local minima and arrive at a faster solution. Figure 4 exhibits the convergence curves that were calculated using the hybrid models that were built. All of the models constructed are compared here in terms of the best and worst convergence behaviors. It can be concluded from the Figure 4 that the best model in terms of convergence was SVM–EO and the worst was SVM–SMA.

Five-fold cross-validation was performed for both the training and testing phases, as shown in Table 4 and Table 5. However, the model was selected based on the lowest RMSE achieved in the testing phase. From Table 5, it can be observed that the SVM–HHO achieved an RMSE of 0.642, which was the lowest among all the cross-validations in the testing phase.

### 4.2. Model Performance

**Figure 5:**
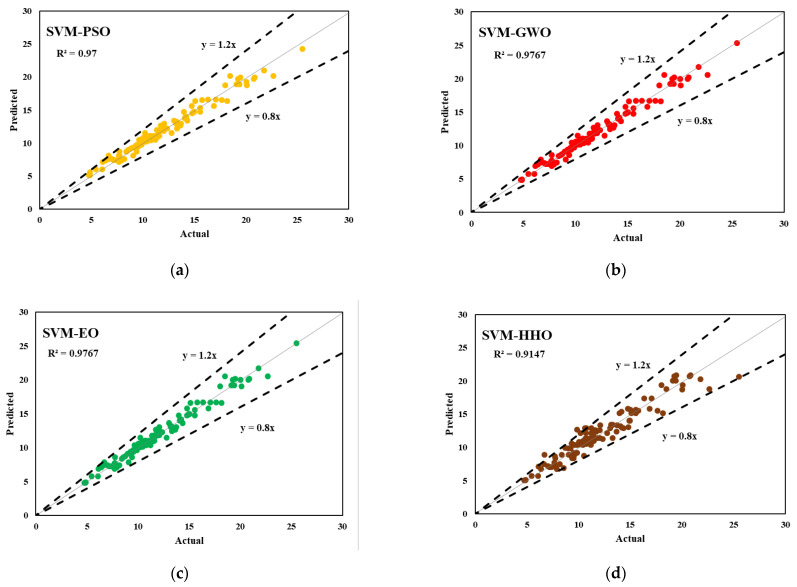

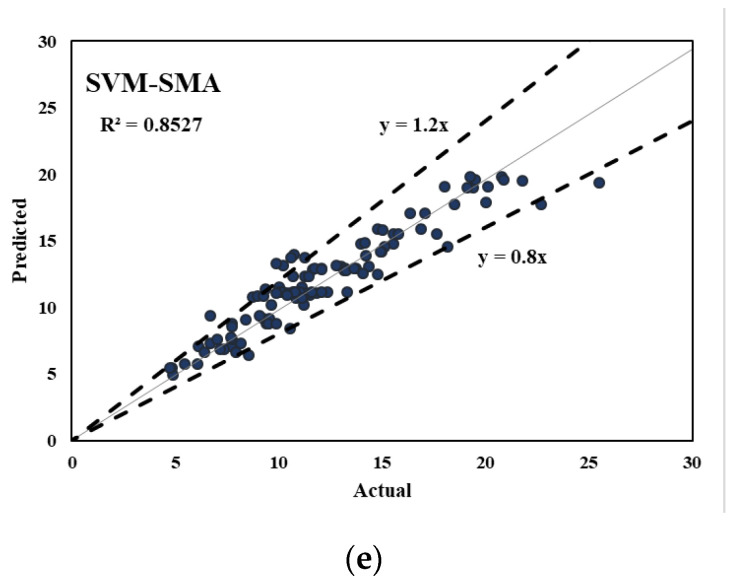
Actual vs. predicted graphs for the training dataset; (**a**) SVM-PSO (**b**) SVM-GWO (**c**) SVM-EO (**d**) SVM-HHO (**e**) SVM-SMA.

**Figure 6:**
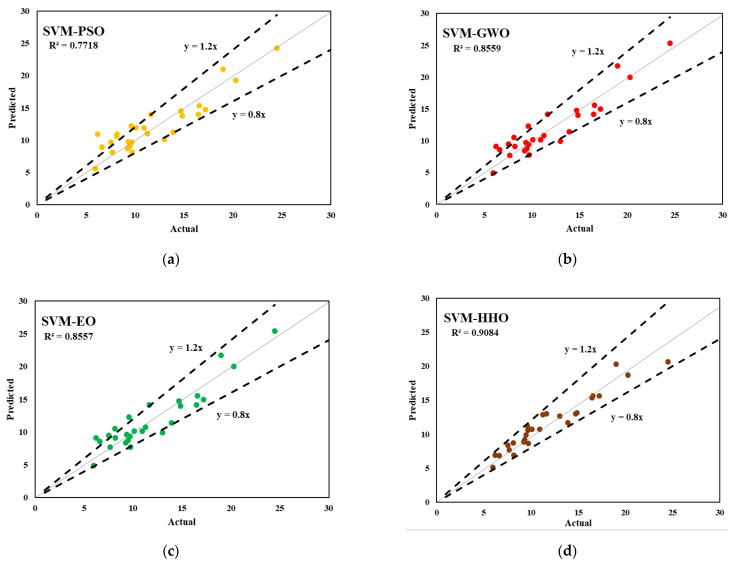

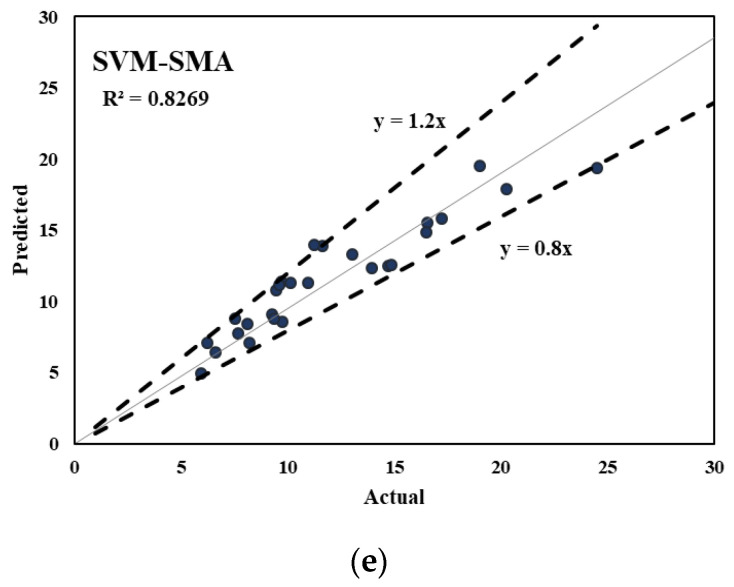
Actual vs. predicted graphs for the testing dataset; (**a**) SVM-PSO (**b**) SVM-GWO (**c**) SVM-EO (**d**) SVM-HHO (**e**) SVM-SMA.

The predictive outcomes of the developed hybrid SVM models for estimating the interfacial bond strength of externally bonded FRP laminates are presented in this section. The performance of the models in predicting the training and testing outputs are reported in Table 6 and Table 7, respectively. It should be noted that each model’s performance with the training subset was used to express the goodness of fit of the constructed models. Based on the experimental results, SVM–GWO and SVM–EO attained the highest R^2^ and the lowest RMSE values (R^2^ = 0.9774 and RMSE = 0.0307), respectively, in the training phase. Among the developed hybrid SVM models, SVM–HHO attained the most desired accuracy, with an R^2^ of 0.9294 and an RMSE of 0.0642 in the training phase. On the other hand, SVM–HHO achieved a prediction performance of R^2^ = 0.9241 and RMSE = 0.0563 in testing. These findings demonstrate that, among the proposed hybrid models, SVM–HHO had good predictive performance in both phases of prediction. In addition, the MAE and WMAPE values of the developed SVM–HHO were determined to be 0.0414 and 0.1169 in training and 0.0520 and 0.1507 in testing, respectively. SVM–GWO and SVM–EO were next-best compared to the above model in training (MAE = 0.0217 and WMAPE = 0.0614), and SVM–SMA was the second-best model (MAE = 0.0647 and WMAPE = 0.1876) in testing. SVM–SMA and SVM–PSO were the worst performing models compared to others in both training and testing. The same results are reported in Figure 5 and Figure 6, which depict the actual versus predicted graphs for both phases.

### 4.3. Taylor Diagrams

**Figure 7:**
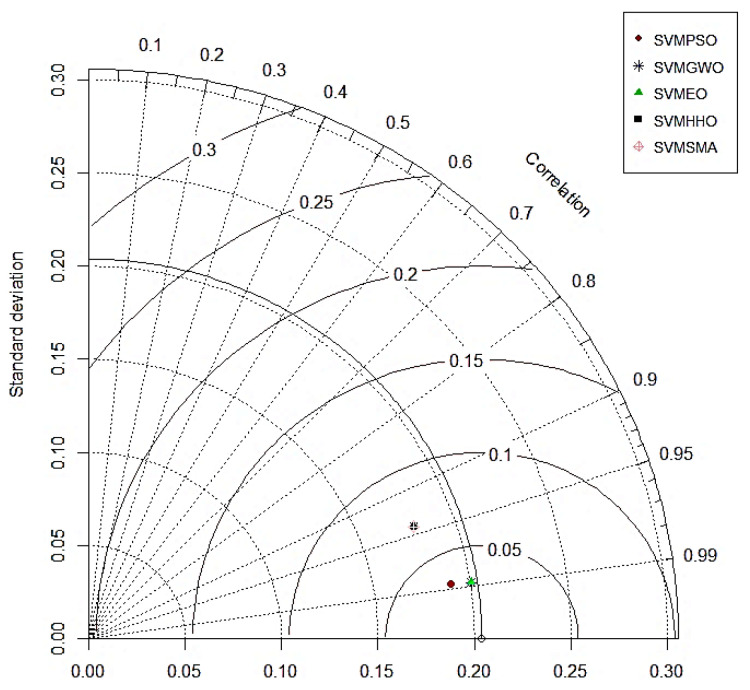
Taylor diagram for the training results.

**Figure 8:**
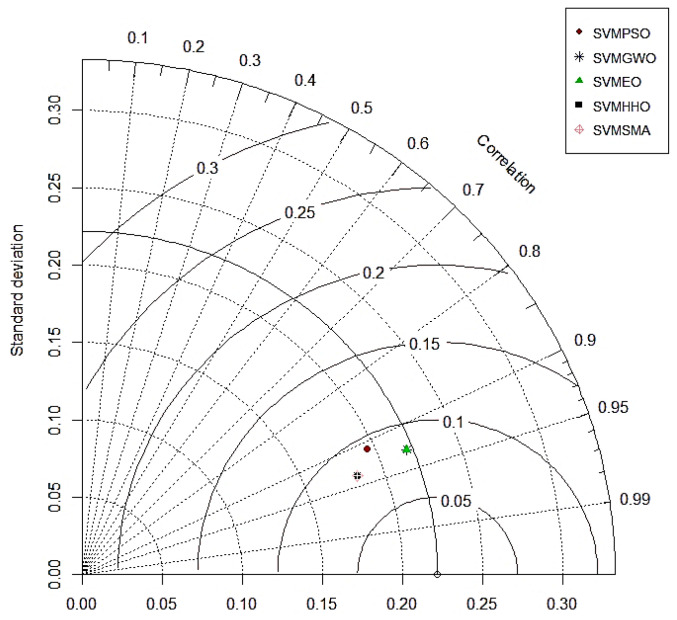
Taylor diagram for the testing results.

As demonstrated in Figure 7 and Figure 8, the Taylor diagram can be used to study the performance of the hybrid SVM models for both the training and testing datasets [134]. The ability of the models to predict the intended output is determined by this diagram. For the relative quantification of the models, we look at three different statistical metrics (RMSE, correlation coefficients and standard deviation ratios). The center RMSE (the distance from the measured point) is taken as the reference point. The standard deviation and correlation coefficient are both set to 1 for the reference model. On the graph, it can be seen that the standard deviation and correlation coefficient values for all five hybrid models were close to 1 for the training phase. It can be concluded from the graph that SVM–SMA had the lowest correlation in training, whereas both SVM–GWO and SVM–EO showed the best performance in the training phase. For the testing dataset, the SVM–HHO model performed the best among all five models, followed by SVM–SMA, SM–GWO and SVM–EO. Hence, it can be concluded that the overall best-performing model was SVM–HHO as it provided good results for both training and testing.

### 4.4. Regression Error Characteristic Curve

**Figure 9:**
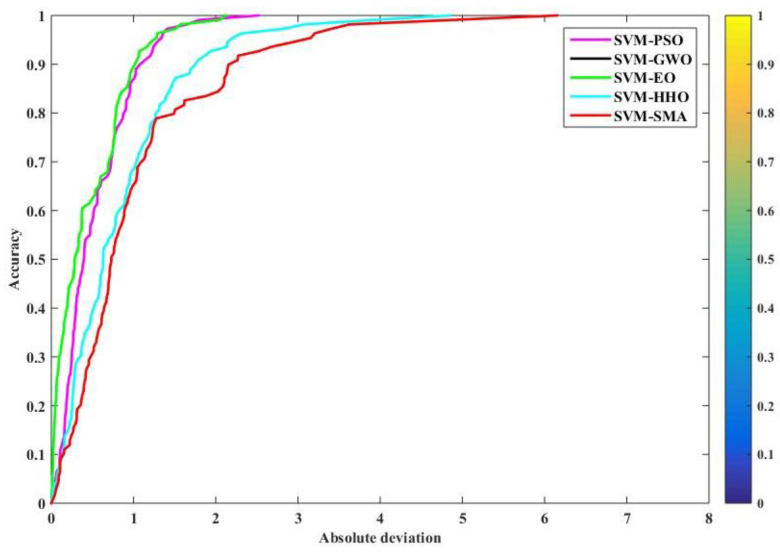
REC curves for training.

**Figure 10:**
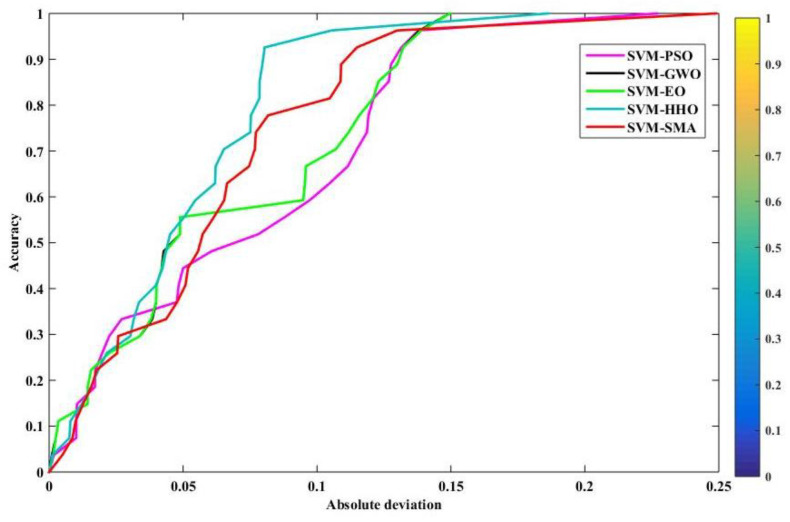
REC curves for testing.

The regression error characteristic (REC) curve plots the graph of error tolerance versus the percentage of predicted points that fall inside the tolerance. The x and y axes show the regression function’s tolerance for errors and its accuracy, respectively. The area over the REC curve (AOC) serves as a good approximation of the projected inaccuracy. The better a model performs, the lower the AOC. Thus, the ROC curve provides a visual representation of a model’s performance that is both quick and precise. Figure 9 and Figure 10 show the REC curves for both stages of the models. A visual interpretation alone shows that SVM–SMA was the least accurate model in the training phase in terms of the accuracy of prediction. We compare the AOC values of different models in order to see how well they function. Table 8 depicts the AOC results. The SVM–GWO and SVM–EO models outperformed the competition during the training phase (with an AOC value of 0.4407); for testing, the SVM–HHO model outperformed the others with an AOC value of 0.0486. For SVM–GWO and SVM–EO, the lines virtually overlap (the black and green lines), and the AOC values were also the same for both training and testing.

## 5. Conclusions

It is relevant to mention that an accurate and trustworthy prediction of the interfacial bond strength of FRP laminates bonded on grooves with concrete prisms will make the construction process more economical. In the current study, a collection of 136 experimental SST datasets with five input parameters was obtained from a literature survey. Some recently developed MOAs were employed in the creation of models using an SVM. Among the models, SVM–GWO and SVM–EO (R^2^ = 0.9774, RSME = 0.0307, WI = 0.9942) were the best performing models in the training stage, followed by SVM–PSO and SVM–HHO; SVM–HHO (R^2^ = 0.9294, RSME = 0.0642, WI = 0.9757) was the best performing model in the testing stage. In addition, SVM–SMA and SVM–PSO were the most underperforming models in the training and testing phases, respectively. The experimental validation of SVM–HHO demonstrates that it has a higher prediction accuracy in both the training and testing stages. These results are significantly better than those obtained from other hybrid SVMs. Based on the experimental outcomes, the proposed SVM–HHO has the potential to assist structural engineers in estimating the ultimate capacity of FRP during the design phase of civil engineering projects. Similar results were also obtained via analysis using Taylor diagrams and REC curves. It is important to note that this study only illustrated the performance efficiency of the models; however, the authors opine that subsequent studies should present detailed parametric and sensitivity analyses for the practical implications of FRP laminates. For now, engineers may use the dataset of the reported study to train the SVM–HHO hybrid model to test new data related to the bond strength of FRP laminates bonded to concrete prisms. However, since this study used hybrid SVM models to predict the bond strength of externally bonded FRP laminates, therefore, the future direction of related work may include the development of an empirical engineering model for a comparative assessment.

## Figures and Tables

**Table 1:** Descriptive statistics of the collected dataset.

Descriptive Statistic	Inputs					Target Variable
	Elastic Modulus of FRP × Thickness of FRP, *E_f_ t_f_*	Width of FRP, *b_f_*	Concrete Compressive Strength, *f_c_*	Width of Groove, *b_g_*	Depth of Groove, *h_g_*	Ultimate Capacity, *p*
Unit	GPa × mm	mm	Mpa	mm	mm	KN
Mean	40.33	46.10	33.72	7.94	10.33	12.05
Standard Error	2.18	1.01	0.73	0.21	0.30	0.37
Median	39.10	50.00	32.70	10.00	10.00	11.11
Mode	78.20	60.00	26.70	10.00	10.00	9.87
Standard Deviation	25.41	11.81	8.49	2.47	3.45	4.32
Sample Variance	645.42	139.52	72.15	6.10	11.93	18.65
Kurtosis	−1.23	−1.49	−1.11	−1.90	−0.88	0.30
Skewness	0.58	−0.13	0.49	−0.36	−0.09	0.80
Range	65.30	30.00	25.50	5.00	10.00	20.73
Minimum	12.90	30.00	22.70	5.00	5.00	4.76
Maximum	78.20	60.00	48.20	10.00	15.00	25.49
Sum	5484.80	6270.00	4585.40	1080.00	1405.00	1638.72
Count	136.00	136.00	136.00	136.00	136.00	136.00
Confidence Level (95.0%)	4.31	2.00	1.44	0.42	0.59	0.73

**Table 2:** Ideal values of different performance parameters.

Indices	R^2^	PI	VAF	WI	RMSE	MAE	RSR	WMAPE
Ideal Value	1	2	100	1	0	0	0	0

**Table 3:** Parametric configuration of hybrid SVM models.

Models	SVM**–**PSO	SVM**–**GWO	SVM**–**EO	SVM**–**HHO	SVM**–**SMA
N_S_	30	30	30	30	30
Itr	200	200	200	200	200
C	0.05	0.10064	0.1	12.5253	71.2704
γ	8.73	100	100	99.3516	71.2704

**Table 4:** Performance of five-fold cross-validation (training phase).

Phase	TR	TR	TR	TR	TR
Models	CV-1	CV-2	CV-3	CV-4	CV-5
SVM–PSO	0.0334	0.0531	0.0561	0.0553	0.0549
SVM–GWO	0.0307	0.0474	0.0499	0.0492	0.0500
SVM–EO	0.0307	0.0474	0.0500	0.0492	0.0500
SVM–HHO	0.0563	0.0571	0.0600	0.0613	0.0550
SVM–SMA	0.0697	0.0696	0.0754	0.0773	0.0691

**Table 5:** Performance of five-fold cross-validation (testing phase).

Phase	TS	TS	TS	TS	TS
Models	CV-1	CV-2	CV-3	CV-4	CV-5
SVM–PSO	0.0936	0.1090	0.0979	0.0688	0.0953
SVM–GWO	0.0829	0.1078	0.0944	0.0688	0.0654
SVM–EO	0.0830	0.1078	0.0942	0.0786	0.0765
SVM–HHO	0.0642	0.1012	0.0981	0.0833	0.0915
SVM–SMA	0.0820	0.0993	0.1029	0.0777	0.0835

**Table 6:** Performance indices for the training dataset.

Indices	SVM**–**PSO	SVM**–**GWO	SVM**–**EO	SVM**–**HHO	SVM**–**SMA
R^2^	0.9763	0.9774	0.9774	0.9241	0.8870
PI	1.9151	1.9229	1.9229	1.7877	1.6949
VAF	97.3227	97.7341	97.7343	92.3648	88.3036
WI	0.9928	0.9942	0.9942	0.9794	0.9661
RMSE	0.0334	0.0307	0.0307	0.0563	0.0697
MAE	0.0260	0.0217	0.0217	0.0414	0.0504
RSR	0.1636	0.1505	0.1505	0.2763	0.3420
WMAPE	0.0730	0.0614	0.0614	0.1169	0.1417

**Table 7:** Performance indices for the testing dataset.

Indices	SVM**–**PSO	SVM**–**GWO	SVM**–**EO	SVM**–**HHO	SVM**–**SMA
R^2^	0.8270	0.8633	0.8631	0.9294	0.8794
PI	1.5185	1.6082	1.6078	1.7690	1.6356
VAF	82.6247	86.0428	86.0258	92.0625	86.6904
WI	0.9480	0.9635	0.9634	0.9757	0.9580
RMSE	0.0936	0.0829	0.0830	0.0642	0.0820
MAE	0.0758	0.0675	0.0676	0.0520	0.0647
RSR	0.4216	0.3737	0.3739	0.2895	0.3694
WMAPE	0.2196	0.1957	0.1958	0.1507	0.1876

**Table 8:** Values of AOC.

Model	AOC Value	
	Training	Testing
SVM–PSO	0.5264	0.0716
SVM–GWO	0.4407	0.0648
SVM–EO	0.4407	0.0648
SVM–HHO	0.8358	0.0486
SVM–SMA	1.0158	0.0601

## Data Availability

The data used in this research have been properly cited and reported in the main text.
